# Trisulfated Heparin Disaccharide (TS-HDS) Antibody-Positive Small Fiber Neuropathy Involving Sweat Gland Nerve Fibers After the Second Moderna Vaccination: A Case Report

**DOI:** 10.7759/cureus.97789

**Published:** 2025-11-25

**Authors:** Josef Finsterer

**Affiliations:** 1 Neurology, Neurology and Neurophysiology Center, Vienna, AUT

**Keywords:** adverse reaction, covid-19 pandemic, moderna, sars-cov-2 vaccination, small fiber neuropathy

## Abstract

SARS-CoV-2 vaccinations (SC2V) may be complicated by small fiber neuropathy (SFN) in individual patients. Furthermore, SC2V-related SFN may be associated with antibodies against FGFR3 or recoverin, but no patient with SC2V-related SFN and elevated trisulfated heparin disaccharide (TS-HDS) antibodies has been described to date.

The patient is a 74-year-old previously healthy woman who experienced temporary side effects after her second Moderna vaccination. One and two months later, more severe symptoms appeared, most of which persisted for the next four years. As sweat gland density was reduced and TS-HDS antibodies were elevated, she was diagnosed with TS-HDS-related SFN, which manifested as regional pain syndrome and autonomic dysfunction, but intravenous immunoglobulins (IVIGs) and naltrexone were ineffective. In addition to SFN, she was also diagnosed with mast cell activation syndrome (MCAS).

This case demonstrates that SC2V with the Moderna vaccine can be complicated by long-lasting TS-HDS antibody-associated SFN and MCAS, which manifest as regional pain, autonomic dysfunction, cognitive impairment, anxiety, tinnitus, myalgia, fatigue, and easy fatigability. IVIGs may be ineffective, so immunosuppressants should be tried.

## Introduction

SARS-CoV-2 vaccines (SC2Vs) are generally well tolerated, but some patients experience mild, moderate, or severe side effects [1]. The frequency of side effects varies considerably between studies supported by vaccine manufacturers [2] and data from real-world practice [3]. Serious side effects include Guillain-Barré syndrome, venous sinus thrombosis, acute disseminated encephalomyelitis (ADEM), and myocarditis [3,4]. Moderate side effects of SC2Vs include headache, brain fog, myositis, and small fiber neuropathy (SFN) [4,5]. SFN results from damage to unmyelinated A-delta and C-fibers, which transmit pain, temperature, and autonomic signals. SFN following SC2V is thought to be immune-mediated and may respond to intravenous immunoglobulin (IVIG) or immunosuppressive agents [6]. Although patients with SC2V-associated SFN and antibodies against fibroblast growth factor receptor 3 (FGFR3) or recoverin [6,7] have been reported, we are not aware of any cases of patients with post-SC2V-associated SFN and elevated trisulfated heparin disaccharide (TS-HDS) antibodies.

## Case presentation

The patient is a 74-year-old previously healthy woman not taking any medications. She developed fever, fatigue, and muscle pain after her second Moderna vaccination (March 2021), which subsided spontaneously after two weeks. In April 2021, one month after vaccination, she developed a new tingling sensation in her right leg, and in May 2021, two months after vaccination, she also noticed pressure in her head (entire skull, including neck, face, eyes, jaw, and ears), facial and jaw pain, and sinus congestion (Table 1). Steroids were prescribed for suspected sinusitis, but they had only a limited effect. In June 2021, she developed tingling, burning, and dysesthesia in both legs and trunk, along with “internal vibrations” and weakness, cramps, and throbbing pain in her legs (Table 1). She also noticed brain fog, stabbing headaches, including in the face and jaw, tinnitus, cognitive deficits, blood pressure instability, tachycardia, extreme fatigue and exercise intolerance, nausea, weight loss, gastrointestinal dysmotility, food intolerances, and balance problems (Table 1). Most of these symptoms persisted throughout 2021 and 2022 (Table 1).

**Table 1:** The index patient’s symptoms at onset and their intensity development over the last four years Grading: 1: mild with no interference in normal daily activities, 2: mild to moderate, 3: moderate, impacts normal daily activities, 4: moderate to severe, 5: severe, unable to complete normal daily activities

Year / Symptoms	2021	2022	2023	2024	2025
Feeling burning pressure in buttocks, thighs, calves	5	5	4	4	4
Tingling in back and arms	4	4	3	3	2
Pulsating pain down backs of legs (worse at night)	5	4	4	3	4
Jolting electric shock sensations	5	3	1	0	0
Muscle cramps	5	4	3	2	2
Squeezing sensation around chest/abdomen	4	3	1	1	1
Internal vibrations	4	4	2	1	0
Pain in teeth and inside of mouth, ear and head	5	5	4	4	4
Tingling lips/around mouth	4	2	1	0	0
Mental fatigue	5	4	3	3	3
Disconnected from surroundings (disassociated)	5	3	2	2	2
Concentration difficulties, inability to stay on task	5	3	3	3	3
Short term memory lapses	4	3	2	1	1
Racing brain, racing thoughts	4	4	2	2	1
Frequent eyestrain and headache, eyes tire easily	4	4	1	1	1
Close up vision difficulty focusing on object	4	3	1	1	1
Muscle twitching in both eyes	5	4	0	0	0
Headache - right side, front and top of head	4	4	4	4	4
Zapping pain in head	5	4	3	1	1
Tinnitus	3	3	3	3	3
Head pressure in face, ears and back of head	5	5	4	4	4
Floating sensation	4	3	2	2	1
Spatial orientation off	5	4	4	4	4
An unusual kind of vertigo (room isn't spinning just "off")	5	4	3	3	3
Pain in right abdomen and back	5	5	3	3	3
change in bowel habits and digestion	5	4	3	4	4
Digestion "messages" inconsistent/irregular	5	4	3	4	4
Nauseous	5	3	1	1	1
Thirsty despite being hydrated	4	2	2	2	2
Abdominal muscle spasms/cramps	5	4	3	1	1
Tire easily, physically and mentally	4	4	4	4	4
Rapid pulse and elevated blood pressure (for no reason)	5	4	3	2	2
Intense symptoms of anxiety (for no reason)	4	2	2	0	0
Feeling of adrenaline surging through body.	5	4	2	0	0
Shaky and can't catch breath (for no reason)	4	2	0	0	0
Extremes in energy levels (varies day to day)	5	3	3	3	3
Low Grade Fever 99.3-99.8 at random times	4	0	0	0	0
Body feels hot without having fever	4	1	0	0	1
Sensation to urinate inconsistent	4	4	2	0	1
Thirsty despite being hydrated	4	2	1	1	1

**Figure 1:**
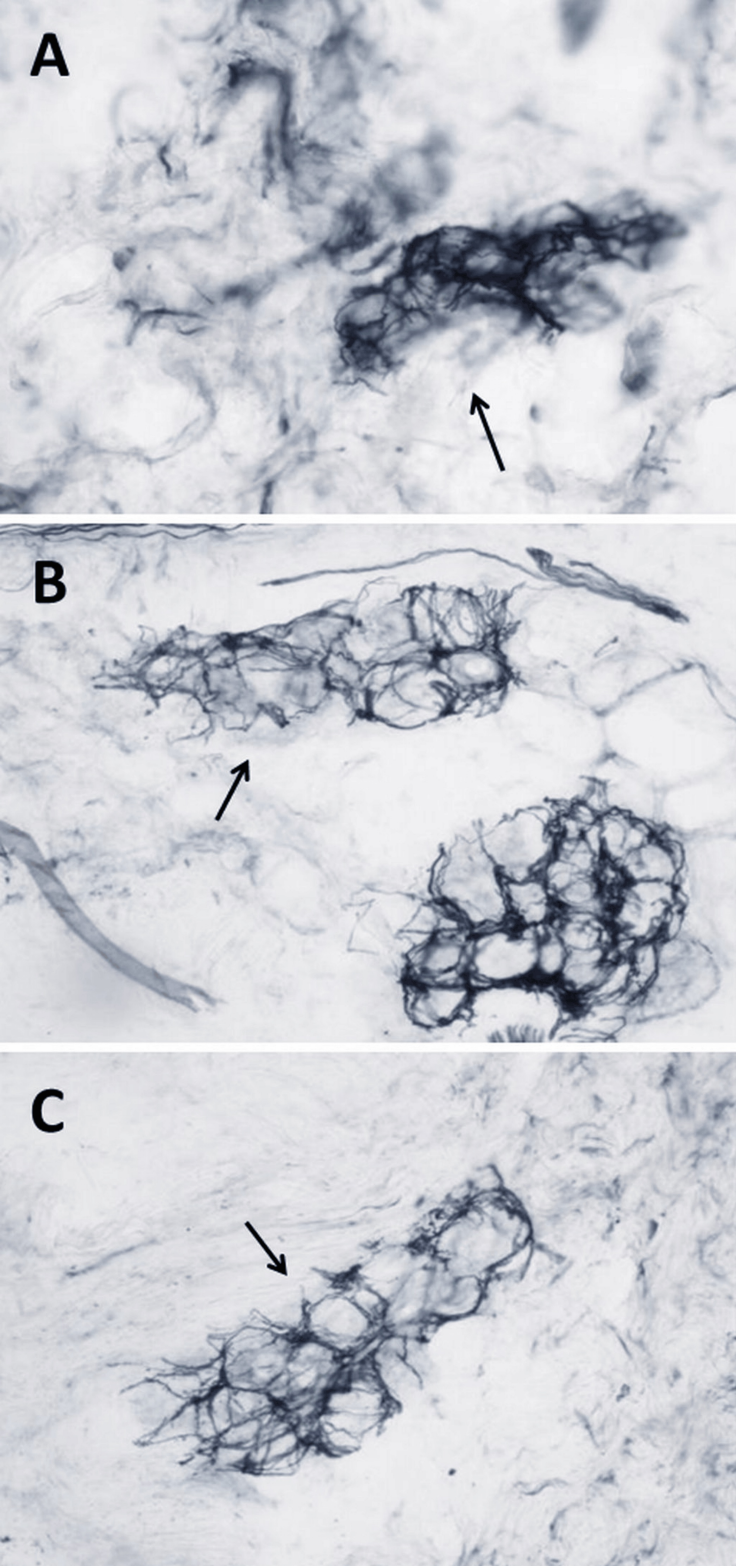
Skin punch biopsy from the right upper thigh (panel A), right lower thigh (panel B), and right calf (panel C) 3 mm, in Zamboni fixative and immunostained with PGP 9.5 according to lab protocols, showing reduced sweat gland nerve fiber density in the right upper thigh (panel A), but normal sweat gland nerve fiber density at the two other locations (panels B, C)

An initial diagnostic examination in February 2022 revealed elevated IgM levels between 290 and 390 mg/dL (n, 26-217 mg/dL) and slightly elevated kappa light chains. This finding led to a diagnosis of monoclonal gammopathy of undetermined significance (MGUS). The MGUS was interpreted as age-related, and MGUS-associated neuropathy was ruled out. The skin biopsy revealed normal intraepidermal nerve fiber density (IENFD) in two locations but decreased sweat gland nerve fiber density in one location (Figure 1). In March 2022, she tested positive for TS-HDS IgM antibodies (29,000 U/mL [n, <10,000 U/mL]) (Table 2). This led to a suspicion of SFN with autonomic dysfunction. In September 2022, mast cell activation syndrome (MCAS) was additionally diagnosed based on symptoms and elevated prostaglandin D2, increased tryptase and N-methylhistamine, and improvement after treatment with mast cell-inhibiting drugs. However, the patient did not tolerate sodium cromolyn, ketotifen, and montelukast. This is why she was switched to intravenous immunoglobulins (IVIGs) (20 g per week from July 2022 to November 2022). IVIGs were discontinued due to side effects such as flu-like symptoms, nausea, headaches, muscle pain, and fatigue. Since November 2022, she has been receiving naltrexone, but this had to be discontinued due to depression and anhedonia.

**Table 2:** Abnormal blood chemical values ALT: alanine transaminase, ANA: anti-nuclear antibodies, AST: aspartate aminotransferase, TS-HDS: trisulfated heparin disaccharide

Parameter	Reference limit	Patient values
ALT	0-45 U/L	58
AST	0-35 U/L	46
IgM	40-230 mg/dL	309
TS-HDS	<10000	29000
Cold agglutinins	≤1:64	1:256
ANA	negative	positive
Prostaglandin D2	35-115 ng/L	228
Kappa light chains	3.3-19.4 mg/L	20.3

Over the course of 2023, the pain and tingling decreased, and gastrointestinal dysmotility gradually improved, but the burning sensation increased (Table 1). A second attempt with subcutaneous immunoglobulins (SCIG) between October and December 2023 had to be discontinued due to the same side effects as with IVIGs. After this second immunoglobulin trial, most of the previously recognized post-vaccination symptoms have persisted (Table 1). A third examination in early 2024 revealed persistently elevated IgM levels, slightly elevated kappa light chains, elevated TS-HDS levels, elevated cold agglutinins, slightly elevated ALT/AST levels, undulating ANA levels, and elevated prostaglandin D2 levels. The remaining blood values were normal. A gastrointestinal biopsy revealed an increase in mast cells, confirming MCAS. Computed tomography (CT) of the brain and neck was unremarkable. MRI of the head and neck without a contrast agent was also inconclusive. The CT scan of the thorax and abdomen with contrast agent was normal, as were the bone marrow biopsy and cerebrospinal fluid (CSF) examination. Most of her symptoms persisted throughout 2024 and continue to this day (September 2025) (Table 1). She was recently taking only fexofenadine (180 mg/d) and famotidine (10 mg/d), but has discontinued even this due to elevated liver enzymes. The patient has not tried an immunosuppressant so far.

## Discussion

The index patient is interesting because she developed SFN associated with TS-HDS antibodies and MCAS one month after receiving the second Moderna vaccination. SFN associated with antibodies against FGFR3 and recoverin has already been reported [6,7], but SFN associated with TS-HDS antibodies has not yet been reported. A 39-year-old man developed regional pain and sensory disturbances a few days after receiving the first BNT162b2 vaccine. Since his IENFD was reduced and antibodies against FGFR3 were elevated, he was diagnosed with immunological SFN [6]. He was treated with IVIGs, which had a positive effect [6]. Even the IENFD improved [6]. Elevated recoverin antibodies were detected in a 47-year-old man with autoimmune retinopathy and SFN after the first dose of BNT162b2 [7]. Most of his symptoms persist to this day, and only glucocorticoids, hyperbaric oxygen therapy, botulinum toxin, inuspheresis, and HELP (heparin-induced extracorporeal LDL precipitation) apheresis showed a temporary positive effect [7]. The significance of TS-HDS is unknown. In a study of 77 patients with elevated TS-HDS antibodies, only 66% had neuropathy, most commonly length-dependent peripheral neuropathy (n=20), length-dependent SFN (n=11), or non-length-dependent SFN (n=7) [8]. Nerve biopsies showed epineural inflammatory cell accumulations in two patients, but no interstitial abnormalities in the remaining seven patients. The majority of IENFDs (7/10), thermoregulatory sweat tests (12/21), and autonomic reflex screenings (27/49) were normal [8]. TS-HDS antibodies are elevated primarily in SFN and are associated with conditions such as dysautonomia [8]. In a retrospective study of 322 patients with SFN, TS-HDS antibodies were elevated in 28% of cases and FGFR3 antibodies in 17% [9]. Although these antibodies can also be found in some people without neuropathy, and their specific role is still being investigated, their presence in SFN is associated with potential immune-mediated damage to small nerve fibers. Damage to small fibers led to symptoms such as neuropathic pain and autonomic dysfunction. It is unknown whether SFN in the index patient is additionally attributable to elevated kappa light chains, but there is evidence that elevated light chains may be responsible for SFN [10].

The second interesting point in this case is that the patient had normal IENFD but reduced sweat gland nerve fiber density. Whether a normal IENFD but reduced sweat gland nerve fiber density indicates SFN is controversial, but there are previous reports of a normal IENFD but reduced sweat gland nerve fiber density [11]. Since sweat glands are innervated by small fibers, patients with normal IENFD but reduced sweat gland nerve fiber density should be diagnosed with SFN.

The third interesting point is the long duration of symptoms. Although some of the symptoms subsided over time without treatment, most symptoms have persisted for over four years to date. Long-lasting symptoms of SC2V-related SFN have been reported [7], suggesting that in some patients, the side effects of SC2V may become a long-term problem known as post-acute COVID vaccination syndrome [7,12]. Post-COVID vaccination syndrome is characterized by long-lasting symptoms similar to long COVID that affect some of the vaccine recipients [4]. The long-lasting symptoms may also be due to the fact that no immunosuppressants have been used to date.

The fourth point is that IVIGs were ineffective. There are few reports on the effect of IVIGs in immune SFN, but most of them described at least some effect [6]. The reason why IVIGs/SCIGs were ineffective in the index patient remains unclear. However, it can be speculated that the pathophysiology of TS-HDS-associated SFN differs from that of other immunological SFN. Different antibodies may target different structures and result in different clinical presentations. 

## Conclusions

This case demonstrates that SC2V with the Moderna vaccine can be associated with long-lasting TS-HDS antibody-associated SFN and MCAS. TS-HDS antibody-associated SFN manifests as regional pain, autonomic dysfunction, cognitive impairment, anxiety, tinnitus, myalgia, and easy fatigability. IVIGs/SCIGs may be ineffective, but further studies need to be conducted to determine whether immunosuppressants are more helpful in preventing serious effects on individual lives.
